# The Book World of Medicine and Science

**Published:** 1905-12-02

**Authors:** 


### The Book World of Medicine and Science.

Gray's Anatomy. Sixteenth Edition. Edited by T. Pickering Pick, F.R.C.S., and Robert Howden, M.A., M.B.C.M. (London: Longmans, Green and Company. 1905. Price 32s. net.)

It is pleasant to know that this standard work shows no sign as yet of failing vitality, for there are few of us who do not regard "Gray's Anatomy" as an old and trusted friend. In this edition numerous additional illustrations have been introduced, and here and there portions of the text have been modified or rewritten to accord with modern requirements. This especially applies to the chapter on embryology. We do not know of any more lucid and accurate account of human embryology in the English language. Additions have been made also to the chapters on the nervous system and the organs of sense. Although containing more substance than its immediate predecessors the present edition is lighter in weight and smaller in volume than these, for a thinner paper has been used. There is no doubt that the volume before us is worthy of the best commendation.

International Clinics. Vol. II. Fifteenth Series. Edited by A. O. J. Kelly, M.D. (London: J. B. Lippincott Company. 1905. Pp. 310. Price 10s. 6d.)

This volume of International Clinics is arranged on practically the same lines as its predecessors. It contains an excellent series of articles written by representative authors, and arranged under the general headings of Treatment, Medicine, Surgery, Gynæcology, Ophthalmology, Rhinology, Physiology, Pathology. The volume is well illustrated and is provided with an ample index.

Organotherapy, or Treatment by Means of Preparations of Various Organs. By H. Batty Shaw, M.D.(Lond.), F.R.C.P. (London: Cassell and Company, Limited. 1905. Illustrated. Pp. 256. Price 6s.)

Following on the advances made in recent years in etiology and symptomatology the treatment of many diseases has made rapid progress. Perhaps the greatest strides have been made in serum treatment and organotherapeutics, and we welcome the third volume of a new series of medical text-books on modern methods of treatment. Many articles and papers on organotherapy have been written and published in England and abroad and Dr. Shaw has done the difficult and somewhat laborious work of bringing together in book-form the essential and most practical points to be culled from an extensive up-to-date literature. More than one-half of the book is given to the thyroid and suprarenal glands. The anatomy, physiology, physiological chemistry, and pathology of these two glands are shortly discussed; and the therapeutics and uses of their extracts are carefully and clearly detailed. Section III. deals in a like manner with the alimentary tract, pancreas and liver; the account of treatment by extracts of these organs is somewhat curtailed. The application in disease of extracts derived from the genito-urinary organs is given in Section IV. It is recommended that, to avoid the influence of suggestion and dislike for such remedies, the patient should be in ignorance of the material used. The final section contains notes on the pituitary body, thymus, spleen, lymphatic glands, marrow, muscle, nerve tissue, and placenta. For purposes of reference Dr. Shaw's book will be invaluable to every up-to-date physician.

